# Early-stage chronic kidney disease as a risk factor for suicide: a nationwide observational cohort study

**DOI:** 10.1007/s40620-025-02219-3

**Published:** 2025-04-09

**Authors:** Hyuk Huh, Kyungdo Han, Minsang Kim, Young Sun Shin, Yeo Jin Yu, Sehyun Jung, Jeong Min Cho, Seong Geun Kim, Semin Cho, Soojin Lee, Eunjeong Kang, Yaerim Kim, Dong Ki Kim, Sehoon Park

**Affiliations:** 1 Department of Internal Medicine, Inje University Busan Paik Hospital, Busan, Korea; 2 Department of Translational Medicine, Seoul National University College of Medicine, Seoul, Korea; 3 Department of Statistics and Actuarial Science, Soongsil University, 369 Sangdo-Ro, Dongjak-Gu, Seoul, 06978 Korea; 4 Department of Internal Medicine, Seoul National University College of Medicine, Seoul, Korea; 5 Department of Internal Medicine, Seoul National University Hospital, 101, Daehak-Ro Jongno-Gu, Seoul, 03080 Korea; 6 Department of Internal Medicine, Gyeongsang National University Hospital, Jinju, Korea; 7 Department of Internal Medicine, Inje University Sanggye Paik Hospital, Seoul, Korea; 8 Department of Internal Medicine, Chung-Ang University College of Medicine, Seoul, Korea; 9 Department of Internal Medicine, Uijeongbu Eulji University Medical Center, Seoul, Korea; 10 Transplantation Center, Seoul National University Hospital, Seoul, Korea; 11 Department of Internal Medicine, Keimyung University School of Medicine, Daegu, Korea; 12 Kidney Research Institute, Seoul National University, Seoul, Korea

**Keywords:** Early-stage chronic kidney disease, Proteinuria, Suicide

## Abstract

**Background:**

Chronic kidney disease (CKD) is associated with poor psychological well-being. Whether early-stage CKD is a risk factor for suicide warrants further research.

**Methods:**

This nationwide, retrospective, cohort study aimed to evaluate the risk of suicide in patients with early-stage CKD and identify the associated risk factors. A total of 3.945,198 individuals aged ≥ 19 years who underwent the 2009 national health screening in South Korea were studied. Among them, 202,291 patients had early-stage CKD (estimated glomerular filtration rate (eGFR) ≥ 30 and < 60 mL/min per 1.73 m^2^ and/or dipstick albuminuria ≥ 1 +). The study outcome was suicide as confirmed by the nationwide death register based on death certificates.

**Results:**

The study population had a mean age of 59 ± 15 years, and 47% were male. We identified 930 suicides (incidence rate, 0.45 per 1000 person-years) in the CKD group and 11,332 suicides (incidence rate, 0.27 per 1000 person-years) in the non-CKD group. Early-stage CKD was significantly associated with an increased risk of suicide in multivariable analysis adjusted for demographic characteristics; lifestyle habits; comorbidities, including diabetes and hypertension; economic status; and depression, bipolar disorder, schizophrenia (hazard ratio, 1.18; 95% confidence interval 1.10‒1.26). Suicide incidence was higher in individuals with proteinuria but preserved kidney function (eGFR > 60 mL/min per 1.73 m^2^ and dipstick albuminuria > 1 +) than in those without CKD.

**Conclusion:**

Healthcare providers may need to examine the mental health of patients with early-stage CKD to prevent suicide.

**Graphical abstract:**

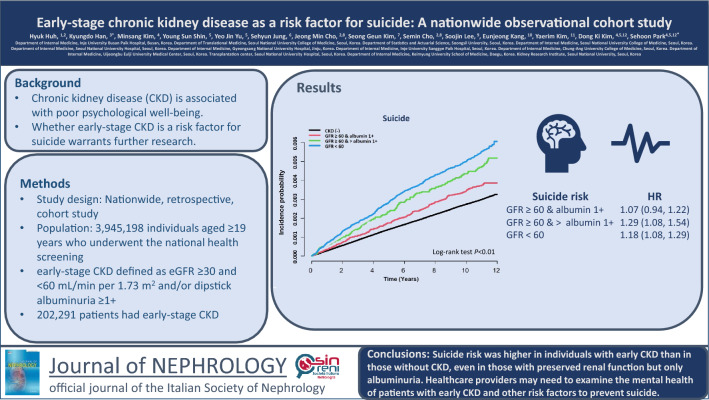

**Supplementary Information:**

The online version contains supplementary material available at 10.1007/s40620-025-02219-3.

## Introduction

The prevalence of chronic kidney disease (CKD) has increased with the expanding aging population and higher rates of diabetes and hypertension [1, 2]. CKD is a global health issue associated with a high mortality risk [3].

CKD diminishes patients’ quality of life and is closely related to poor psychological well-being and depressive mood [4]. Although general medical care may have improved the physical prognosis of patients with CKD, additional efforts are needed to address the mental health issues associated with CKD. Kidney injury can have systemic effects, including on the brain. Studies investigating the mechanisms underlying this crosstalk have been performed [5]. A common pathway involving endothelial cell injury may contribute to the neurological complications observed in patients with CKD, which can include cognitive decline, vascular disorders, anxiety, sleep disorders, and depression [6, 7]. Furthermore, patients with severe mental illnesses, such as schizophrenia and bipolar disorder, are at increased risk of mortality from associated medical comorbidities [8]. In addition to depression affecting patients with advanced CKD requiring dialysis, depression and suicidal ideation have been reported to increase even in the early stages of CKD [9]. Therefore, further studies are needed to investigate whether early-stage CKD is associated with a higher risk of suicide in contemporary society and to identify the risk factors associated with this potentially preventable cause of death.

In this study, we hypothesized that the risk of suicide may be higher among individuals with early-stage CKD, including those without an overt reduction in the estimated glomerular filtration rate (eGFR) but with albuminuria. The study used the national claims database of South Korea, including general health examination results, to study the population that clinicians commonly encounter during routine clinical visits. The study aimed to assess the risk of suicide in the early-stage CKD population and identify risk factors for such events.

## Methods

### Study setting

This retrospective cohort study utilized data from the National Health Insurance Service (NHIS) database, including demographic assessments, lifestyle habits, anthropometric measurements, and laboratory tests [10]. Data from nationwide health screenings and the composition of the NHIS database have been previously described [10, 11]. The NHIS database in South Korea provides comprehensive data on healthcare usage, health screenings, demographics, and mortality for the entire population. It includes information on health behaviors and bio-clinical variables within the screening database, as well as data on inpatient/outpatient usage (diagnosis, service received) and prescription records (drug code, days prescribed, daily dosage) in a healthcare utilization database. The nationwide health screening was provided free of charge, and the quality of health screening was controlled by the NHIS. Health screening is offered to the general population both annually and biennially. The collected health screening data were linked to a nationwide claims database. As the NHIS provides services to all citizens in South Korea, the nationwide claims database is well suited for population-scale studies.

We identified adults who underwent health screening at the age of ≥ 19 years in 2009. We collected information on demographic characteristics, including age; sex; body mass index (BMI); waist circumference; smoking history; alcohol intake (> 0 g of alcohol intake per day); regular physical activity (moderate-intensity physical activity ≥ 5 days or vigorous-intensity physical activity ≥ 3 days per week); low-income status (lower quartile of the nation); comorbidities, including hypertension, diabetes mellitus, and dyslipidemia; anthropometric measurements, including systolic blood pressure (BP) and diastolic BP; and laboratory results, including dipstick albuminuria, serum glucose, total cholesterol, high-density lipoprotein cholesterol (HDL-C), and low-density lipoprotein cholesterol (LDL-C). Depression, bipolar disorder, and schizophrenia were defined as a diagnosis made by a psychiatrist using specific ICD-10 codes: F32; major depressive disorder, single episode, F33; major depressive disorder, recurrent, F20; Schizophrenia, F31; bipolar affective disorder, respectively [12, 13].

### CKD as exposure and suicide as study outcome

The presence of CKD was defined as an eGFR of < 60 mL/min per 1.73 m^2^, calculated using the Modification of Diet in Renal Disease equation, or the presence of dipstick albuminuria ≥ 1 + during health screening. Among the health assessments indicative of CKD, we excluded individuals with end-stage kidney disease (ESKD) or an eGFR of < 30 mL/min per 1.73 m^2^. ESKD was defined by insurance coverage codes for kidney replacement therapy including hemodialysis, peritoneal dialysis, and transplantation in the NHIS database. Finally, we defined early-stage CKD as CKD stage 1–2 with eGFR > 60 mL/min per 1.73 m^2^ and albuminuria, or CKD stage 3 with eGFR 30–60 mL/min per 1.73 m^2^ regardless of albuminuria. The study outcome was suicide, based on the claims database, which included nationwide mortality events collected from death certificates.

### Statistical analysis

The risk of outcomes was evaluated using Cox regression analysis. The cumulative incidence of outcomes was presented using the Kaplan–Meier survival curve. Multivariate models were adjusted for age, BMI, smoking status, alcohol consumption, regular exercise, history of comorbidities (including hypertension, diabetes mellitus, dyslipidemia, atrial fibrillation, cancer), and depression, bipolar disorder, schizophrenia. We used an interaction test to evaluate significant differences between subgroups. Interactions between variables were determined using the interaction term *P* values in the multivariable model. All statistical analyses were conducted using SAS. Two-sided *P* values < 0.05 were considered statistically significant.

## Results

### Baseline characteristics

After applying the exclusion criteria, among 4,234,415 individuals who underwent the national health screening, 3,945,198 were selected for this study (Supplemental Fig. 1). Among the participants, 202,291 individuals were diagnosed with early CKD (stage 3). Individuals with CKD were further categorized as follows: eGFR > 60 mL/min per 1.73 m^2^ and dipstick albuminuria 1 + (*n* = 60,833), eGFR > 60 mL/min per 1.73 m^2^ and dipstick albuminuria > 1 + (*n* = 26,852), eGFR 30–60 mL/min per 1.73 m^2^ and dipstick albuminuria negative (n = 104,791), eGFR 30–60 mL/min per 1.73 m^2^ and dipstick albuminuria 1 + (5259), and eGFR 30–60 mL/min per 1.73 m^2^ and dipstick albuminuria > 1 + (*n* = 4556). Based on the income distribution of CKD patients divided into four quartiles, 44,239 patients (21.86%) were in the 1st quartile, 38,610 (19.08%) in the 2nd quartile, 49,182 (24.31%) in the 3rd quartile, and 70,260 (34.73%) in the 4th quartile. In terms of BMI, 72,268 (35.72%) had a BMI between 25 and 30, and 11,382 (5.62%) had a BMI greater than 30. Depending on the presence or absence of CKD and whether they died by suicide, differences in baseline characteristics were observed (Table 1). Individuals who died by suicide in the CKD group were not evidently poor and did not drink more heavily. However, individuals who died by suicide in the CKD were older and had a higher prevalence of diabetes mellitus, hypertension, and depression than individuals without CKD.

**Table 1:** Baseline characteristics according to CKD and suicide

N	CKD (−)		CKD ( +)		*P* value
	Suicide (−)	Suicide (+)	Suicide (−)	Suicide (+)	
	3,731,575	11,332	201,361	930	
Age (years)	46.5 ± 13.7	51.6 ± 14.8	59.0 ± 15.0	64.0 ± 1	
< 40	1,206,296 (32.3)	2.574 (22.7)	21,738 (10.8)	60 (6.5)	< 0.001
40–64	2,094,059 (56.1)	6.075 (53.6)	96,830 (48.1)	323 (34.7)	
≥ 65	431,220 (11.6)	2.683 (23.7)	82,793 (41.1)	547 (58.8)	
Sex (male)	2,058,153 (55.2)	8.708 (76.8)	94,406 (46.9)	627 (67.4)	< 0.001
Income (quartile)					
Q1	807,110 (21.6)	2,726 (24.1)	44,029 (21.9)	210 (22.6)	< 0.001
Q2	848,666 (22.7)	2,565 (22.6)	38,421 (19.1)	189 (20.3)	
Q3	1,001,237 (26.8)	2,961 (26.1)	48,958 (24.3)	224 (24.1)	
Q4	1,074,562 (28.8)	3,080 (27.2)	69,953 (34.7)	307 (33.0)	
Smoke					
Non	2,209,749 (59.2)	4,651 (41.0)	135,050 (67.1)	490 (52.7)	< 0.001
Ex-smoker	530,221 (14.2)	1,830 (16.2)	29,701 (14.8)	182 (19.6)	
Current	991,605 (26.6)	4,851 (42.8)	36,610 (18.2)	258 (27.7)	
Drink					
Non	1,900,822 (50.9)	4,898 (43.2)	132,024 (65.6)	583 (62.7)	< 0.001
Mild	1,530,860 (41.0)	4,895 (43.2)	56,561 (28.1)	252 (27.1)	
Heavy	299,893 (8.0)	1,539 (13.6)	12,776 (6.3)	95 (10.2)	
Exercise	675,441 (18.1)	2,279 (20.1)	38,004 (18.9)	180 (19.4)	< 0.001
DM	295,436 (7.9)	1,454 (12.8)	47,378 (23.5)	288 (31.0)	< 0.001
HTN	899,999 (24.1)	3,795 (33.5)	107,971 (53.6)	586 (63.0)	< 0.001
Dyslipidemia	644,661 (17.3)	2,183 (19.3)	67,775 (33.7)	312 (33.6)	< 0.001
Depression	267,032 (7.2)	1,896 (16.7)	26,393 (13.1)	220 (23.7)	< 0.001
Bipolar disorder	4,782 (0.13)	84 (0.74)	380 (0.19)	4 (0.43)	< 0.001
Schizophrenia	4,417 (0.12)	74 (0.65)	322 (0.16)	2 (0.22)	< 0.001
Height (cm)	164.1 ± 9.2	165.2 ± 8.6	160.4 ± 9.5	161.3 ± 9.5	< 0.001
Weight (kg)	64.0 ± 11.6	64.1 ± 11.1	63.0 ± 11.8	62.8 ± 11.4	< 0.001
BMI (kg/m^2^)	23.7 ± 3.2	23.4 ± 3.2	24.4 ± 3.4	24.0 ± 3.4	< 0.001
WC (cm)	80.1 ± 9.1	81.4 ± 8.6	83.0 ± 9.4	84.4 ± 9.0	< 0.001
SBP (Hg)	122.2 ± 14.9	124.9 ± 15.4	127.8 ± 17.1	129.8 ± 17.7	< 0.001
DBP (Hg)	76.2 ± 10.0	77.7 ± 10.2	78.4 ± 11.0	79.3 ± 11.4	< 0.001
Glucose (mg/dL)	96.7 ± 22.7	100.4 ± 29.0	107.9 ± 37.3	113.2 ± 43.5	< 0.001
TC (mg/dL)	194.8 ± 36.6	194.3 ± 37.7	201.4 ± 42.0	197.5 ± 43.2	< 0.001
HDL (mg/dL)	55.7 ± 23.2	55.5 ± 27.3	54.2 ± 23.3	53.5 ± 20.5	< 0.001
LDL (mg/dL)	113.4 ± 38.7	110.4 ± 42.3	117.0 ± 40.8	111.6 ± 40.2	< 0.001
eGFR (ml/min per 1.73 m^2^)	94.4 ± 16.4	92.5 ± 15.9	69.4 ± 22.3	66.4 ± 21.1	< 0.001
TG (mg/dL)	111.7 (111.7–111.8)	124.1 (122.7–125.4)	129.8 (129.4–130.1)	138.7 (133.8–143.9)	< 0.001

Data are presented as mean ± standard deviation, or n (%)

*BMI* body mass index, *CKD* chronic kidney disease, *DM* diabetes mellitus, HTN hypertension, *SBP* systolic blood pressure, *DBP* diastolic blood pressure, *HDL* high-density lipoprotein cholesterol, *LDL* low-density lipoprotein cholesterol, *eGFR* estimated glomerular filtration rate, *TC* total cholesterol, *WC* waist circumference, *TG* triglycerides

### Suicide risk among patients with early CKD

**Fig. 1:**
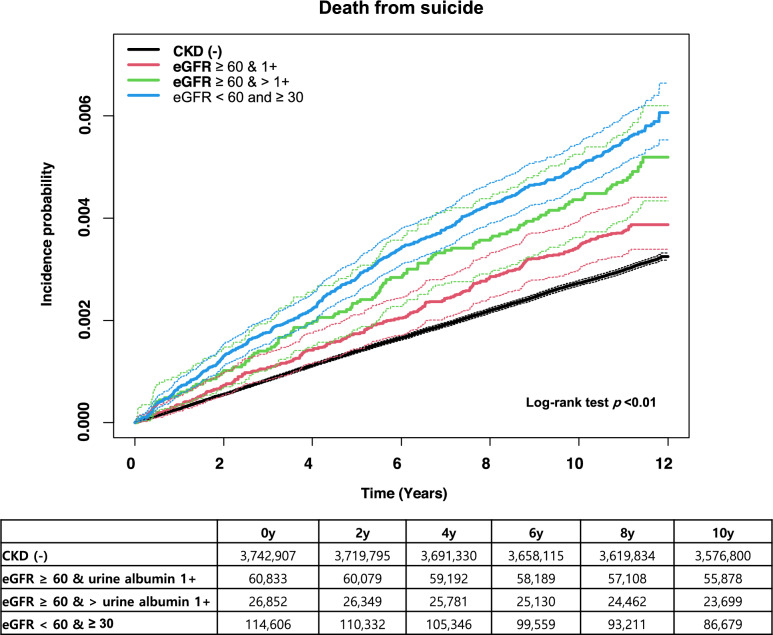
Cumulative incidence of suicide according to CKD. The x-axis indicates time (years), and the y-axis indicates the cumulative incidence (percentage) of suicide during the observation period. Survival curves were stratified by eGFR and proteinuria (black, non-CKD; red, eGFR > 60 ml/min per 1.73 m^2^ and dipstick albuminuria 1 + ; green, eGFR > 60 ml/min per 1.73 m^2^ and dipstick albuminuria > 1 + ; and blue, eGFR 30–60 ml/min per 1.73 m^2^ and dipstick albuminuria negative). CKD, chronic kidney disease; eGFR, estimated glomerular filtration rate

The median follow-up duration was 11.3 (IQR, 11.1–11.57) years. The suicide incidence rate was 0.45 per 1000 person-years in the CKD group, which was higher than the 0.27 per 1000 person-years in the non-CKD group. Regarding eGFR and proteinuria, the association between CKD and the risk of suicide was investigated. Compared with the non-CKD group, the risk of suicide was increased among individuals with an eGFR of > 60 mL/min per 1.73 m^2^ and dipstick albuminuria > 1 + (HR, 1.29; 95% CI 1.08–1.54), as well as among those with an eGFR of 30–60 mL/min per 1.73 m^2^ (Fig. 1). This result remained significant in multivariable analysis adjusted for demographic characteristics, lifestyle habits, comorbidities, and depression, bipolar disorder, schizophrenia (hazard ratio [HR], 1.18; 95% confidence interval [95% CI], 1.10‒1.26) (Table 2). In this model, older age, diabetes mellitus, hypertension, current smoking, heavy drinking, and depression were significant risk factors for suicide (Supplemental Table 1).

**Table 2:** Suicide risk among patients with CKD

	*N*	Event	Incidence rate	Model 1^a^	Model 2^b^	Model 3^c^	Model 4^d^	Model 5^e^
			(/1000 person-years)	HR (95% CI)	HR (95% CI)	HR (95% CI)	HR (95% CI)	HR (95% CI)
CKD (−)	3,742,907	11,332	0.27	1 (reference)	1 (reference)	1 (reference)	1 (reference)	1 (reference)
CKD ( +)	202,291	930	0.45	1.63 (1.53, 1.75)	1.20 (1.12, 1.29)	1.19 (1.11, 1.27)	1.16 (1.09, 1.25)	1.18 (1.10, 1.26)
CKD (−)	3,742,907	11,332	0.27	1 (reference)	1 (reference)	1 (reference)	1 (reference)	1 (reference)
eGFR > 60 mL/min per 1.73 m^2^ and dipstick albuminuria 1 +								
	60,833	224	0.34	1.24 (1.09, 1.42)	1.12 (0.98, 1.28)	1.07 (0.94, 1.23)	1.07 (0.94, 1.22)	1.08 (0.94, 1.23)
eGFR > 60 mL/min per 1.73 m^2^ and dipstick albuminuria > 1 +								
	26,852	126	0.44	1.61 (1.35, 1.92)	1.38 (1.16, 1.65)	1.29 (1.08, 1.54)	1.29 (1.08, 1.54)	1.30 (1.09, 1.56)
eGFR 30 ≤ and < 60 mL/min per 1.73 m^2^								
	114,606	580	0.51	1.86 (1.71, 2.02)	1.20 (1.10, 1.31)	1.21 (1.11, 1.32)	1.18 (1.08, 1.29)	1.20 (1.10, 1.31)

^a^Model 1: Crude

^b^Model 2: Adjusted for age and sex

^c^Model 3: Model 2 adjusted for BMI, income level, smoking, drinking, diabetes, hypertension, dyslipidemia, and physical activity

^d^Model 4: Model 3 adjusted for depression

^e^Model 5: Model 4 adjusted for bipolar disorder, schizophrenia

CKD, chronic kidney disease; eGFR, estimated glomerular filtration rate; HR, hazard ratio

### Suicide risk among patients with early CKD without depression

Considering that depression is a major risk factor for suicide, the association between CKD and suicide risk varied significantly according to the presence of depression (*P* for interaction, 0.03). We performed a subgroup analysis after stratification by age, BMI, and the presence of depression/schizophrenia/bipolar disorder (Supplemental Table 2). Although there was only a small number of individuals with depression, bipolar disorder, or schizophrenia, CKD was not associated with the risk of suicide. However, CKD was a significant risk factor for suicide in those without diagnosed depression, bipolar disorder, or schizophrenia.

### Factors associated with suicide in the early-stage CKD population

The average age of patients who died by suicide in the CKD group was 64.0 ± 14 years, and 67.4% were male (Supplemental Table 3). They also had a higher prevalence of hypertension and diabetes. In the CKD group, older individuals and male patients were at a higher risk of suicide (Table 3). Current smokers had a significantly higher risk of suicide than never smokers, and a low-income status was a risk factor for suicide. Higher BMI was associated with a lower risk of suicide. Depression and migraines were significant risk factors for suicide in the CKD group. In a multivariable model according to eGFR, overweight and obesity were observed to have a protective effect against suicide compared to normal weight in CKD with preserved kidney function (eGFR > 60 mL/min per 1.73 m^2^) (Supplemental Table 4). While chronic medical illnesses, such as diabetes and newly diagnosed hypertension, were significant risk factors for suicide in this group. However, in CKD with an eGFR of 30–60 mL/min per 1.73 m^2^, regular exercise was associated with a lower risk of suicide, while migraine was related to a higher risk of suicide.

**Table 3:** Factors associated with suicide in the CKD population

	*N*	Suicide	Incidence rate (/1000 person-years)	Univariable HR (95% CI)	Multivariable^a^ HR (95% CI)
Age				1.03 (1.03, 1.04)	1.04 (1.03, 1.04)
Sex					
Male	95,033	627	0.65	1 (reference)	1 (reference)
Female	107,258	303	0.27	0.41 (0.36, 0.47)	0.39 (0.33, 0.47)
Income					
Q1	44,239	210	0.46	1 (reference)	1 (reference)
Q2	38,610	189	0.47	1.02 (0.84, 1.24)	1.08 (0.87, 1.31)
Q3	49,182	224	0.44	0.96 (0.79, 1.16)	0.93 (0.77, 1.12)
Q4	70,260	307	0.43	0.94 (0.79, 1.12)	0.80 (0.67, 0.95)
* P* for trend				0.35	< 0.01
BMI (kg/m^2^)					
18.5 <	6,453	34	0.57	1.11 (0.78, 1.58)	1.25 (0.87, 1.78)
18.5–23	62,803	324	0.51	1 (reference)	1 (reference)
23–25	49,385	219	0.43	0.84 (0.71, 0.99)	0.77 (0.65, 0.92)
25–30	72,268	315	0.41	0.81 (0.70, 0.95)	0.75 (0.64, 0.88)
> 30	11,382	38	0.31	0.62 (0.44, 0.86)	0.69 (0.49, 0.97)
* P* for trend				< 0.01	< 0.01
Smoke					
Non	135,540	490	0.35	1 (reference)	1 (reference)
Ex	29,883	182	0.60	1.73 (1.46, 2.05)	1.08 (0.88, 1.31)
Current	36,868	258	0.68	1.97 (1.69, 2.29)	1.57 (1.31, 1.89)
*P* for trend				< 0.01	< 0.01
Drink					
Non	132,607	583	0.43	1 (reference)	1 (reference)
Mild	56,813	252	0.42	0.97 (0.84, 1.12)	0.85 (0.72, 1.01)
Heavy	12,871	95	0.71	1.65 (1.32, 2.04)	1.17 (0.93, 1.49)
*P* for trend				< 0.01	0.87
Regular exercise					
No	164,107	750	0.44	1 (reference)	1 (reference)
Yes	38,184	180	0.45	1.01 (0.85, 1.18)	0.93 (0.79, 1.10)
Diabetes mellitus					
Normal	103,875	419	0.38	1 (reference)	1 (reference)
IFG	50,750	223	0.42	1.11 (0.94, 1.30)	0.95 (0.81, 1.12)
New	11,871	66	0.55	1.44 (1.11, 1.86)	1.20 (0.93, 1.57)
< 5 years	13,675	86	0.64	1.67 (1.33, 2.11)	1.26 (1.00, 1.61)
≥ 5 years	22,120	136	0.66	1.71 (1.41, 2.07)	1.18 (0.96, 1.44)
*P* for trend				< 0.01	0.02
Hypertension					
Normal	38,706	116	0.28	1 (reference)	1 (reference)
Pre-HTN	55,028	228	0.39	1.40 (1.12, 1.76)	1.17 (0.93, 1.46)
New onset	19,868	93	0.45	1.63 (1.24, 2.15)	1.22 (0.92, 1.61)
Control	57,122	316	0.56	2.01 (1.63, 2.49)	1.24 (0.98, 1.56)
Uncontrol	31,567	177	0.57	2.05 (1.62, 2.59)	1.29 (1.01, 1.66)
*P* for trend				< 0.01	0.049
Total cholesterol (mg/dL)					
< 200	80,061	398	0.49	1 (reference)	1 (reference)
< 240	54,143	220	0.39	0.80 (0.68, 0.94)	0.85 (0.72, 1.01)
≥ 240	22,366	84	0.36	0.74 (0.59, 0.94)	0.83 (0.65, 1.05)
Med ( +)	45,721	228	0.49	1.01 (0.86, 1.18)	0.89 (0.72, 1.10)
*P* for trend				0.717	0.079
HDL (< 40/50 mg/dL or taken lipid lowering agent)					
No	112,621	523	0.44	1 (reference)	1 (reference)
Yes	89,670	407	0.45	1 (0.88, 1.14)	0.94 (0.79, 1.12)
Depression					
No	175,678	710	0.39	1 (reference)	1 (reference)
Yes	26,613	220	0.84	2.17 (1.86, 2.52)	2.08 (1.77, 2.43)
Psoriasis					
No	197,590	895	0.44	1 (reference)	1 (reference)
Yes	4,701	35	0.75	1.7 (1.21, 2.38)	1.38 (0.98, 1.93)
Atrial fibrillation					
No	197,113	899	0.44	1 (reference)	1 (reference)
Yes	5,178	31	0.70	1.56 (1.09, 2.23)	1.05 (0.73, 1.51)
Migraine					
No	178,351	794	0.43	1 (reference)	1 (reference)
Yes	23,940	136	0.56	1.29 (1.08, 1.55)	1.25 (1.04, 1.51)
Cancer					
No	196,086	893	0.44	1 (reference)	1 (reference)
Yes	6,205	37	0.65	1.46 (1.05, 2.03)	1.13 (0.81, 1.57)

^a^Multivariable model was adjusted for all variables included in the table. The variables age and BMI are continuous, and hazard ratios are reported per 1-year in age and per 1 kg/m^2^ in BMI

*BMI* body mass index, *CKD* chronic kidney disease, *HTN* hypertension, *HR* hazard ratio, *IFG* impaired fasting glucose, *HDL* high-density lipoprotein cholesterol, *LDL* low-density lipoprotein cholesterol

## Discussion

This nationwide, population-based cohort study showed that early-stage CKD was associated with the risk of suicide. The risk of suicide was higher in patients with early-stage CKD (eGFR, 30–60 ml/min per 1.73 m^2^) than in those without CKD. Even in patients with CKD with preserved renal function but only albuminuria, a higher risk of suicide was observed than that in individuals without CKD. CKD remained a significant risk factor for suicide even after adjustment for the traditional risk factors, including current smoker, heavy drinker, chronic medical illness and depression, bipolar disorder, schizophrenia. Furthermore, this study demonstrated that risk and protective factors for suicide may differ based on kidney function. These results suggest that healthcare providers need to pay attention to the mental health of patients with early-stage CKD to reduce preventable deaths.

The association between depression and kidney function has been extensively investigated. The prevalence of major depressive episodes is 21% in patients with CKD, regardless of stage [14]. A recent meta-analysis reported that depressive symptoms are significantly associated with kidney function [15]. The presence of depression in patients with CKD is associated with increased hospitalization and dialysis initiation, independent of kidney disease severity [16]. Depression is also associated with increased hospitalization and mortality rates in patients with ESKD who require dialysis [17, 18]. Furthermore, advanced CKD and ESKD are known to increase the risk of suicide. An analysis of the United States end-stage renal disease program revealed that the risk of suicide was significantly increased compared to that in the general population (1.84 of the overall standardized incidence ratio for suicide) [19]. Given that dialysis causes acute stress reactions in patients with ESKD, they may experience increased depression and suicidal ideation [20, 21]. Additionally, loss of social roles and physical independence could contribute to a high risk of suicide at the beginning of dialysis [22].

Additional large-scale studies are necessary to investigate whether depression is associated with the future risk of suicide, particularly in patients with early-stage CKD. In a case–control study, Liu et al. reported an elevated risk of suicide in patients with CKD, including those on dialysis, even after adjusting for depressive disorders [23]. This result is consistent with our finding that the risk of suicide was significantly higher in patients with CKD without depression. The strengths of the current study include that we (1) identified that individuals with early-stage CKD had a higher risk of suicide; (2) observed this in a large-scale nationwide cohort that included information on causes of death from death certificates; and (3) investigated specific risk factors associated with the risk of suicide in the CKD population.

Our findings suggest that patients with early-stage CKD may experience undetected suicidal ideation, even if the patients have not been diagnosed with depression, bipolar disorder, or schizophrenia. Although a clear mechanism has not yet been established, multiple factors may be responsible for the increased risk of suicide among patients with CKD. A Mendelian randomization study showed a causal link between psychological well-being and kidney function [24]. Thus, a poor sense of well-being in patients with CKD may lead to a high risk of suicide. Chronic medical illnesses closely related to CKD, including diabetes, malignancy, and hypertension, may contribute to a higher risk of suicide [25–27]. Furthermore, the association between CKD and suicide remained significant after adjusting for baseline economic status; however, patients with chronic medical diseases, including CKD, might experience poor socioeconomic status afterwards, leading to a high risk of suicide [28–30].

The risk factors associated with suicide in patients with CKD include various clinical demographic factors: old age, male sex, lower body mass index, poor income, smoking, diabetes mellitus, hypertension, presence of depression, and migraine. Most risk factors have also been reported in the general population; therefore, paying particular attention to patients with CKD who have such high-risk characteristics may be helpful in preventing suicide. On the other hand, the risk factors differed according to eGFR in early-stage CKD: preserved eGFR (≥ 60 mL/min/1.73 m^2^), diabetes, hypertension, or smoking were more prominent risk factors than reduced eGFR (< 60 mL/min/1.73 m^2^). The risk factors associated with suicide in patients with reduced eGFR included migraine and a lack of regular exercise. The fact that patients with reduced eGFR may be more likely to have an underlying illness could have led to this finding. This is because physical discomfort, such as migraines, or a lack of regular exercise may be a more important determinant of the mental health of patients with kidney dysfunction.

Our results should be interpreted considering the current suicide epidemiology in South Korea. Unfortunately, among Organization for Economic Cooperation and Development (OECD) countries, South Korea has the highest suicide rate (26.0 per 100,000 persons). Suicide is the 10th leading cause of death worldwide and the 5th leading cause of death in Korea. The suicide rate has not decreased over the past 10 years. Thus, the risk of suicide in the CKD population may have been accentuated by the high overall incidence of suicide in Korea.

This study has some limitations. First, potential selection bias remains, as the study included patients who underwent a general health examination. This may include healthier individuals because of healthy volunteer bias. However, this suggests that healthcare providers may need to focus on the mental health of those with kidney dysfunction, even if they are not critically ill, and skip general health examinations. Second, because this was a single-nation study, the association between suicide risk and CKD may differ in other countries. Third, confounders may have been present due to the observational nature of the study.

In conclusion, early-stage CKD is associated with the risk of suicide, even in patients with CKD with preserved renal function but only albuminuria. To reduce preventable deaths, healthcare providers in nephrology need to pay attention to the mental health status of patients with early-stage CKD and other risk factors.

## Supplementary Information

Below is the link to the electronic supplementary material.

Supplementary file1 (DOCX 43 KB)

Supplementary file2 (PDF 45 KB)

## Data Availability

This study used data from the National Health Insurance Service of Republic of Korea. Data are available from the Korean National Health Insurance Sharing Service. Researchers who wish to access the data can apply at https://nhiss.nhis.or.kr/bd/ay/bdaya001iv.do.
