# Supplementary material for: Early-stage chronic kidney disease as a risk factor for suicide: a nationwide observational cohort study
Source: J Nephrol. 2025 Apr 9;38(3):989–98. doi: 10.1007/s40620-025-02219-3 (PMC12165974; doi:10.1007/s40620-025-02219-3)
Supplement: Supplementary file 1 — Supplementary file1 (DOCX 43 KB) [file 40620_2025_2219_MOESM1_ESM.docx]

**Supplemental Table 1.** Multivariable model for suicide risk

|  | **N** | **Event** | **Incidence rate** | **Model 1^a^** | **Model 2^b^** | **Model 3^c^** | **Model 4^d^** |
| --- | --- | --- | --- | --- | --- | --- | --- |
|  |  |  | (/1,000 person-years) | HR (95% CI) | HR (95% CI) | HR (95% CI) | HR (95% CI) |
| CKD (-) | 3,742,907 | 11,332 | 0.27 | 1 (reference) | 1 (reference) | 1 (reference) | 1 (reference) |
| CKD (+) | 202,291 | 930 | 0.45 | 1.63 (1.53, 1.75) | 1.20 (1.12, 1.29) | 1.19 (1.11, 1.27) | 1.16 (1.09, 1.25) |
| CKD (-) | 3,742,907 | 11,332 | 0.27 | 1 (reference) | 1 (reference) | 1 (reference) | 1 (reference) |
| eGFR >60 mL/min per 1.73 m^2^ and dipstick albuminuria 1+ | | | |  |  |  |  |
|  | 60,833 | 224 | 0.34 | 1.24 (1.09, 1.42) | 1.12 (0.98, 1.28) | 1.07 (0.94, 1.23) | 1.07 (0.94, 1.22) |
| eGFR >60 mL/min per 1.73 m^2^ and dipstick albuminuria >1+ | | | |  |  |  |  |
|  | 26,852 | 126 | 0.44 | 1.61 (1.35, 1.92) | 1.38 (1.16, 1.65) | 1.29 (1.08, 1.54) | 1.29 (1.08, 1.54) |
| eGFR 30≤ and <60 mL/min per 1.73 m^2^ | | | |  |  |  |  |
|  | 114,606 | 580 | 0.51 | 1.86 (1.71, 2.02) | 1.20 (1.10, 1.31) | 1.21 (1.11, 1.32) | 1.18 (1.08, 1.29) |
| Age |  |  |  |  | 1.04 (1.03, 1.04) | 1.03 (1.03, 1.04) | 1.03 (1.03, 1.03) |
| Sex (female) |  |  |  |  | 0.33 (0.32, 0.34) | 0.4 (0.38, 0.42) | 0.37 (0.35, 0.39) |
| Income |  |  |  |  |  |  |  |
| Q1 |  |  |  |  |  | 1 (reference) | 1 (reference) |
| Q2 |  |  |  |  |  | 0.94 (0.89, 0.99) | 0.93 (0.89, 0.98) |
| Q3 |  |  |  |  |  | 0.83 (0.79, 0.87) | 0.82 (0.78, 0.86) |
| Q4 |  |  |  |  |  | 0.73 (0.70, 0.77) | 0.72 (0.68, 0.76) |
| Diabetes |  |  |  |  |  | 1.24 (1.18, 1.31) | 1.23 (1.16, 1.29) |
| Hypertension |  |  |  |  |  | 1.19 (1.14, 1.24) | 1.17 (1.12, 1.22) |
| Dyslipideima |  |  |  |  |  | 1.00 (0.96, 1.05) | 0.97 (0.93, 1.02) |
| Smoke |  |  |  |  |  |  |  |
| Non |  |  |  |  |  | 1 (reference) | 1 (reference) |
| Ex-smoker |  |  |  |  |  | 0.96 (0.93, 1.04) | 0.98 (0.92, 1.04) |
| Current |  |  |  |  |  | 1.65 (1.57, 1.73) | 1.65 (1.57, 1.73) |
| Drink |  |  |  |  |  |  |  |
| Non |  |  |  |  |  | 1 (reference) | 1 (reference) |
| Mild |  |  |  |  |  | 0.99 (0.95, 1.03) | 0.98 (0.92, 1.04) |
| Heavy |  |  |  |  |  | 1.26 (1.18, 1.34) | 1.30 (1.22, 1.38) |
| Regular exercises |  |  |  |  |  | 1.01 (0.97, 1.06) | 1.01 (0.97, 1.06) |
| BMI |  |  |  |  |  | 0.94 (0.93, 0.94) | 0.94 (0.93, 0.94) |
| Depression |  |  |  |  |  |  | 2.64 (2.53, 2.77) |

^a^Model 1: Crude

^b^Model 2: Adjusted for age and sex

^c^Model 3: Model 2 adjusted for BMI, income level, smoking, drinking, diabetes, hypertension, dyslipidemia, and physical activity

^d^Model 4: Model 3 adjusted for depression

CKD, chronic kidney disease; eGFR, estimated glomerular filtration rate; HR, hazard ratio.

**Supplemental Table 2.** Subgroup analysis

|  |  | **N** | **Suicide** | **Duration** | **Incidence rate**  (/1,000 person-years) | **Model^*^**  HR (95% CI) | ***P* for interaction** |
| --- | --- | --- | --- | --- | --- | --- | --- |
| Age <65 | CKD (-) | 3,309,004 | 8,649 | 37,166,693 | 0.23 | 1 (reference) | 0.78 |
|  | CKD (+) | 118,951 | 383 | 1,315,944 | 0.29 | 1.12 (1.01, 1.24) |  |
| Age ≥65 | CKD (-) | 433,903 | 2,683 | 4,415,866 | 0.61 | 1 (reference) |  |
|  | CKD (+) | 83,340 | 547 | 773,168 | 0.71 | 1.14 (1.04, 1.25) |  |
| Sex, male | CKD (-) | 2,066,861 | 8,708 | 22,821,298 | 0.38 | 1 (reference) | 0.24 |
|  | CKD (+) | 95,033 | 627 | 959,274 | 0.65 | 1.13 (1.04, 1.23) |  |
| Sex, female | CKD (-) | 1,676,046 | 2,624 | 18,761,261 | 0.14 | 1 (reference) |  |
|  | CKD (+) | 107,258 | 303 | 1,129,838 | 0.27 | 1.24 (1.10, 1.39) |  |
| BMI <25 | CKD (-) | 2,531,938 | 7,950 | 28,096,615 | 0.28 | 1 (reference) | 0.42 |
|  | CKD (+) | 118,641 | 577 | 1,206,503 | 0.48 | 1.14 (1.04, 1.24) |  |
| BMI ≥25 | CKD (-) | 1,210,969 | 3,382 | 13,485,943 | 0.25 | 1 (reference) |  |
|  | CKD (+) | 83,650 | 353 | 882,608 | 0.40 | 1.21 (1.08, 1.35) |  |
| Depression (-) | CKD (-) | 3,473,979 | 9,436 | 38,652,676 | 0.24 | 1 (reference) | 0.03 |
|  | CKD (+) | 175,678 | 710 | 1,828,637 | 0.39 | 1.22 (1.12, 1.32) |  |
| Depression (+) | CKD (-) | 268,928 | 1,896 | 2,929,883 | 0.65 | 1 (reference) |  |
|  | CKD (+) | 26,613 | 220 | 260,475 | 0.84 | 1.02 (0.88, 1.17) |  |
| Bipolar disorder (-) | CKD (-) | 3,738,041 | 11,248 | 41,532,808 | 0.27 | 1 (reference) | 0.10 |
|  | CKD (+) | 201,907 | 926 | 2,082,693 | 0.45 | 1.19 (1.11, 1.27) |  |
| Bipolar disorder (+) | CKD (-) | 4,866 | 84 | 52,378 | 1.60 | 1 (reference) |  |
|  | CKD (+) | 384 | 4 | 3,791 | 1.06 | 0.51 (0.19, 1.39) |  |
| Schizophrenia (-) | CKD (-) | 3,738,416 | 11,258 | 41,538,917 | 0.27 | 1 (reference) | 0.06 |
|  | CKD (+) | 201,967 | 928 | 2,083,369 | 0.45 | 1.19 (1.11, 1.27) |  |
| Schizophrenia (+) | CKD (-) | 4,491 | 74 | 46,269 | 1.60 | 1 (reference) |  |
|  | CKD (+) | 324 | 2 | 3,116 | 0.64 | 0.31 (0.08, 1.29) |  |

^*^Model: Adjusted for age, sex, BMI, income level, smoking, drinking, diabetes, hypertension, dyslipidemia, and physical activity, depression

Abbreviation: CKD, chronic kidney disease; eGFR, estimated glomerular filtration rate; BMI, body mass index

**Supplemental Table 3.** Comparison of baseline characteristics in CKD

|  | **Suicide (-)** | **Suicide (+)** | ***P*-value** |
| --- | --- | --- | --- |
| N | 201,361 | 930 |  |
| Age (year) | 59.0 ± 15.0 | 64.0 ± 14 | <0.01 |
| <40 | 21,738 (10.8) | 60 (6.5) |  |
| 40-64 | 96,830 (48.1) | 323 (34.7) |  |
| ≥65 | 82,793 (41.1) | 547 (58.8) |  |
| Sex (male) | 944,06 (46.9) | 627 (67.4) | <0.01 |
| Income (quartile) |  |  | 0.62 |
| Q1 | 44,029 (21.9) | 210 (22.6) |  |
| Q2 | 38,421 (19.1) | 189 (20.3) |  |
| Q3 | 48,958 (24.3) | 224 (24.1) |  |
| Q4 | 69,953 (34.7) | 307 (33.0) |  |
| BMI (kg/m^2^) |  |  | 0.039 |
| < 18.5 | 6,419 (3.2) | 34 (3.7) |  |
| 18.5-23 | 62,479 (31.0) | 324 (34.8) |  |
| 23-25 | 49,166 (24.4) | 219 (23.6) |  |
| 25-30 | 71,953 (35.7) | 315 (33.9) |  |
| ≥30 | 11,344 (5.6) | 38 (4.1) |  |
| Smoke |  |  | <0.01 |
| Non | 135,050 (67.1) | 490 (52.7) |  |
| Ex | 29,701 (14.8) | 182 (19.6) |  |
| Current | 36,610 (18.2) | 258 (27.7) |  |
| Drink |  |  | <0.01 |
| Non | 132,024 (65.6) | 583 (62.7) |  |
| Mild | 56,561 (28.1) | 252 (27.1) |  |
| Heavy | 12,776 (6.3) | 95 (10.2) |  |
| Regular exercise | 38,004 (18.9) | 180 (19.4) | 0.71 |
| DM | 47,378 (23.5) | 288 (31.0) | <0.01 |
| HTN | 107,971 (53.6) | 586 (63.0) | <0.01 |
| Dyslipidemia | 67,775 (33.7) | 312 (33.6) | 0.94 |
| Depression | 26,393 (13.1) | 220 (23.7) | <0.01 |
| Bipolar | 380 (0.19) | 4 (0.43) | <0.01 |
| Schizophrenia | 322 (0.16) | 2 (0.22) | <0.01 |
| Psoriasis | 4,666 (2.3) | 35 (3.8) | <0.01 |
| Atrial fibrillation | 5,147 (2.6) | 31 (3.3) | 0.13 |
| Migraine | 23,804 (11.8) | 136 (14.6) | <0.01 |
| Cancer | 6,168 (3.1) | 37 (4.0) | 0.11 |
| Height (cm) | 160.4 ± 9.5 | 161.3 ± 9.5 | <0.01 |
| Weight (kg) | 63.0 ± 11.8 | 62.8 ± 11.4 | 0.59 |
| BMI (kg/m^2^) | 24.4 ± 3.42 | 24.0 ± 3.4 | <0.01 |
| WC (cm) | 83.0 ± 9.4 | 84.4 ± 9.0 | <0.01 |
| SBP (Hg) | 127.8 ± 17.1 | 129.8 ± 17.7 | <0.01 |
| DBP (Hg) | 78.4 ± 10.9 | 79.3 ± 11.4 | 0.02 |
| Glucose (mg/dL) | 107.9 ± 37.3 | 113.2 ± 43.5 | <0.01 |
| TC (mg/dL) | 201.4 ± 42.0 | 197.5 ± 43.2 | <0.01 |
| HDL (mg/dL) | 54.2 ± 23.3 | 53.5 ± 20.5 | 0.37 |
| LDL (mg/dL) | 117.0 ± 40.8 | 111.6 ± 40.2 | <0.01 |
| eGFR (ml/min per 1.73m^2^) | 69.4 ± 22.3 | 66.4 ± 21.1 | <0.01 |
| TG (mg/dL) | 129.8 (129.4 - 130.1) | 138.7 (133.8 - 143.9) | <0.01 |

Data are presented as the mean ± standard deviation, or n (%)

Abbreviation: BMI, body mass index; CKD, chronic kidney disease; DM, diabetes mellitus; HTN, hypertension; SBP, systolic blood pressure; DBP, diastolic blood pressure; HDL. high-density lipoprotein cholesterol; LDL, low-density lipoprotein cholesterol; eGFR, estimated glomerular filtration rate; TC, total cholesterol; WC, waist circumstance; TG, triglyceride

**Supplemental Table 4.** Risk factors for suicide in CKD according to eGFR

|  | **N** | **Suicide** | **Incidence rate**  (/1,000 person-years) | **Multivariable**  **eGFR ≥60**  HR (95% CI) | **Multivariable**  **eGFR < 60**  HR (95% CI) |
| --- | --- | --- | --- | --- | --- |
| Age |  |  |  | 1.02 (1.01, 1.03) | 1.06 (1.05, 1.07) |
| Sex |  |  |  |  |  |
| Male | 95,033 | 627 | 0.65 | 1 (reference) | 1 (reference) |
| Female | 107,258 | 303 | 0.27 | 0.50 (0.36, 0.69) | 0.35 (0.28, 0.43) |
| Income |  |  |  |  |  |
| Q1 | 44,239 | 210 | 0.46 | 1 (reference) | 1 (reference) |
| Q2 | 38,610 | 189 | 0.47 | 1.10 (0.82, 1.47) | 1.01 (0.78, 1.32) |
| Q3 | 49,182 | 224 | 0.44 | 0.76 (0.56, 1.03) | 1.04 (0.82, 1.32) |
| Q4 | 70,260 | 307 | 0.43 | 0.76 (0.56, 1.01) | 0.80 (0.64, 0.99) |
| *P* for trend | |  |  |  | <0.01 |
| BMI (kg/m^2^) |  |  |  |  |  |
| 18.5< | 6,453 | 34 | 0.57 | 1.29 (0.76, 2.17) | 1.12 (0.69, 1.81) |
| 18.5 - 23 | 62,803 | 324 | 0.51 | 1 (reference) | 1 (reference) |
| 23 - 25 | 49,385 | 219 | 0.43 | 0.68 (0.51, 0.90) | 0.86 (0.69, 1.07) |
| 25 - 30 | 72,268 | 315 | 0.41 | 0.55 (0.42, 0.71) | 0.93 (0.76, 1.13) |
| >30 | 11,382 | 38 | 0.31 | 0.52 (0.31, 0.87) | 0.79 (0.49, 1.26) |
| *P* for trend | |  |  |  | <0.01 |
| Smoke |  |  |  |  |  |
| Non | 135,540 | 490 | 0.35 | 1 (reference) | 1 (reference) |
| Ex | 29,883 | 182 | 0.60 | 1.06 (0.74, 1.53) | 1.13 (0.89, 1.42) |
| Current | 36,868 | 258 | 0.68 | 2.10 (1.56, 2.83) | 1.23 (0.95, 1.59) |
| *P* for trend | | |  |  |  |
| Drink |  |  |  |  |  |
| Non | 132,607 | 583 | 0.43 | 1 (reference) | 1 (reference) |
| Mild | 56,813 | 252 | 0.42 | 0.82 (0.63, 1.06) | 0.89 (0.72, 1.10) |
| Heavy | 12,871 | 95 | 0.71 | 1.27 (0.92, 1.75) | 0.944 (0.63, 1.41) |
| *P* for trend | |  |  |  | 0.37 |
| Regular exercise | |  |  |  |  |
| No | 164,107 | 750 | 0.44 | 1 (reference) | 1 (reference) |
| Yes | 38,184 | 180 | 0.45 | 1.26 (0.98, 1.61) | 0.79 (0.63, 0.98) |
| Diabetes mellitus | |  |  |  |  |
| Normal | 103,875 | 419 | 0.38 | 1 (reference) | 1 (reference) |
| IFG | 50,750 | 223 | 0.42 | 0.98 (0.74, 1.31) | 0.96 (0.78, 1.17) |
| New | 11,871 | 66 | 0.55 | 1.55 (1.07, 2.26) | 0.99 (0.67, 1.46) |
| <5 years | 13,675 | 86 | 0.64 | 1.51 (1.02, 2.23) | 1.18 (0.87, 1.61) |
| ≥5 years | 22,120 | 136 | 0.66 | 1.64 (1.17, 2.29) | 1.02 (0.79, 1.33) |
| *P* for trend | | |  |  |  |
| Hypertension |  |  |  |  |  |
| Normal | 38,706 | 116 | 0.28 | 1 (reference) | 1 (reference) |
| Pre HTN | 55,028 | 228 | 0.39 | 1.21 (0.87, 1.68) | 1.15 (0.84, 1.58) |
| New onset | 19,868 | 93 | 0.45 | 1.49 (1.01, 2.21) | 1.00 (0.67, 1.50) |
| Control | 57,122 | 316 | 0.56 | 1.22 (0.83, 1.79) | 1.25 (0.93, 1.68) |
| Uncontrol | 31,567 | 177 | 0.57 | 1.41 (0.93, 2.11) | 1.27 (0.92, 1.76) |
| *P* for trend | |  |  |  | 0.17 |
| Total cholesterol (mg/dL) | | |  |  |  |
| <200 | 80,061 | 398 | 0.49 | 1 (reference) | 1 (reference) |
| <240 | 54,143 | 220 | 0.39 | 0.96 (0.73, 1.25) | 0.82 (0.65, 1.01) |
| ≥240 | 22,366 | 84 | 0.36 | 1.07 (0.75, 1.52) | 0.70 (0.50, 0.98) |
| Med (+) | 45,721 | 228 | 0.49 | 0.85 (0.58, 1.23) | 0.94 (0.73, 1.20) |
| *P* for trend | |  |  |  | 0.53 |
| HDL (<40/50mg/dL or Med) | | |  |  |  |
| No | 112,621 | 523 | 0.44 | 1 (reference) | 1 (reference) |
| Yes | 89,670 | 407 | 0.45 | 1.08 (0.80, 1.46) | 0.87 (0.70, 1.08) |
| Depression |  |  |  |  |  |
| No | 175,678 | 710 | 0.39 | 1 (reference) | 1 (reference) |
| Yes | 26,613 | 220 | 0.84 | 2.49 (1.87, 3.30) | 1.91 (1.58, 2.31) |
| Psoriasis |  |  |  |  |  |
| No | 197,590 | 895 | 0.44 | 1 (reference) | 1 (reference) |
| Yes | 4,701 | 35 | 0.75 | 1.46 (0.82, 2.60) | 1.33 (0.88, 2.02) |
| Atrial fibrillation | | |  |  |  |
| No | 197,113 | 899 | 0.44 | 1 (reference) | 1 (reference) |
| Yes | 5,178 | 31 | 0.70 | 1.24 (0.58, 2.64) | 0.98 (0.65, 1.48) |
| Migraine |  |  |  |  |  |
| No | 178,351 | 794 | 0.43 | 1 (reference) | 1 (reference) |
| Yes | 23,940 | 136 | 0.56 | 1.13 (0.78, 1.64) | 1.31 (1.06, 1.63) |
| Cancer |  |  |  |  |  |
| No | 196,086 | 893 | 0.44 | 1 (reference) | 1 (reference) |
| Yes | 6,205 | 37 | 0.65 | 1.72 (0.96, 3.09) | 0.96 (0.64, 1.44) |

^a^Multivariable model was adjusted for all variables included in the table

Abbreviation: BMI, body mass index; CKD, chronic kidney disease; HTN, hypertension; HR, hazard ratio; IFG, impaired fasting glucose; HDL. high-density lipoprotein cholesterol; LDL, low-density lipoprotein cholesterol;
