# Supplementary figures and images for: Early-stage chronic kidney disease as a risk factor for suicide: a nationwide observational cohort study
Source: J Nephrol. 2025 Apr 9;38(3):989–98. doi: 10.1007/s40620-025-02219-3 (PMC12165974; doi:10.1007/s40620-025-02219-3)

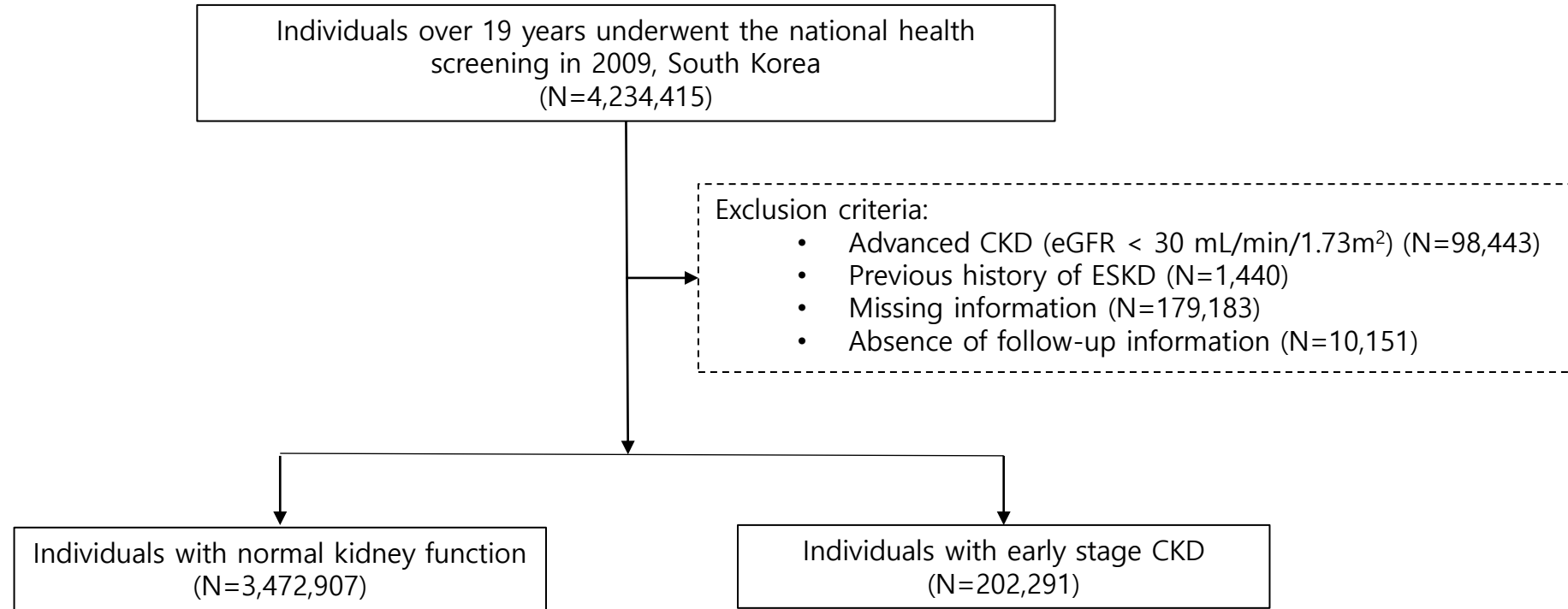

Supplement: Supplementary file 2 — Supplementary file2 (PDF 45 KB) [file 40620_2025_2219_MOESM2_ESM.pdf]
